# The Micromechanical Measurement of Photothermal Expansion †

**DOI:** 10.3390/mi17101129

**Published:** 2026-09-28

**Authors:** Luca Quaroni

**Affiliations:** Department of Physical Chemistry and Electrochemistry, Faculty of Chemistry, Jagiellonian University, 30-387 Cracow, Poland; luca.quaroni@uj.edu.pl; Tel.: +48-12-6862520

**Keywords:** photothermal effect, photothermal expansion, optomechanical sensor, scanning probe, micro engine

## Abstract

The measurement of photothermal expansion via the deflection of a cantilevered probe has attracted attention as a tool for the investigation of condensed-phase materials. In the present work, I provide a simple theoretical analysis of signal generation by this micromechanical detection mechanism using the classical description of constrained thermal expansion. I relate signal intensity to the thermomechanical properties of the sample and the probe, and their contact geometry for the case of gradual excitation. The results support the general applicability of this configuration for the measurement of photothermal expansion and provide guidelines for optimization of the signal. I also provide a conceptual framework to analyze the measurement by describing it as a thermodynamic engine cycle, comprising a series of quasi-static processes. The engine is intrinsically inefficient when assessed in its capacity to convert energy into work, and the energy derived from light absorption is dispersed into the environment as heat. However, the thermodynamic efficiency is not a critical performance parameter when considering the main purpose of this mechanism, which is analytical.

## 1. Introduction

The photothermal effect converts the light absorbed by a material into heat, leading to a local increase in temperature and pressure, and a corresponding volume expansion. The effect was first observed by Bell, who reported the associated sound emission [1]. Over the years, multiple measurement configurations have been conceived to monitor photothermal expansion and to develop it into a tool for the investigation of materials [2]. Among these configurations, the use of atomic force microscopy (AFM) instrumentation is receiving increasing attention. Expansion is monitored via the deflection of the cantilever of an AFM probe in contact with the sample, providing local information about local light absorption and heating [3,4].

The magnitude of the photothermal expansion tracks the absorption coefficient of the material and is wavelength dependent, a property which has been exploited for spectroscopic analysis. The spatial resolution of the measurement is independent from the wavelength of the exciting radiation, creating the opportunity to avoid the limits set by diffraction. This characteristic is particularly appealing in the mid-infrared (mid-IR) spectral region, where the relatively long wavelength (μ~2.5–25 µm) corresponds to a diffraction limited resolution of the order of a few micrometers. The feasibility of this micromechanical detection scheme for photothermal expansion in the mid-IR was demonstrated using continuous wave (CW) light sources [3]. The experiments relied on cantilever deflection to detect photothermal expansion of the order of the nanometer, easily accessible to standard AFM instrumentation (Figure 1). As in standard AFM operation, the deflection was quantified by the movement of a laser probe beam on a four-quadrant detector. These measurements also provided a mechanically detected interferogram when using light modulated by the Michelson interferometer of a Fourier - Transform Infrared (FTIR) optical bench, although no spectrum could be obtained by its Fourier Transform. The method was later developed into a technique for spectroscopic analysis using the higher power pulsed emission from a free-electron laser as a light source [4]. The AFM-based detection scheme was commercialized with benchtop pulsed laser sources, extending its accessibility to a larger pool of researchers. Different names are used to describe the various incarnations of this configuration, among which common ones are AFM-IR and Photothermal-Induced Resonance (PTIR) [4,5]. The former has been popularized with commercial products and has seen more widespread use.

In this work, I distinguish between two different modes of excitation in photothermal experiments, which shall be called gradual and impulsive. Gradual excitation involves the use of a continuous wave (CW) laser, or a thermal light source. In such a configuration, the sample is exposed to continuous illumination, and the temperature of the sample increases progressively, until illumination is interrupted, for example, by chopping the beam, or until the rate of heating is matched by the rate of cooling and a steady state is achieved [3,6]. It is taken to be a defining feature of gradual excitation that the cantilever deflection must track the expansion and contraction of the sample without loss of contact from the tip. A slow sinusoidal modulation of light intensity can also be considered a form of gradual excitation as long as the latter condition is met. In the case of impulsive excitation, the sample is rapidly heated by a laser pulse. The impulse excites resonant modes of the cantilever, giving rise to exponentially decaying oscillations, the amplitude of which is used as a proxy for light absorption [4]. In the most general case of impulsive excitation, the oscillations of the cantilever do not track the expansion and contraction of the sample in phase, except under resonant conditions [7]. In the latter case, the complex movement of the cantilever resonating according to its normal modes drives the oscillation of the laser beam on the four-quadrant detector, instead of the simple deflection.

Despite the rising interest in spectroscopic applications of the technique, aspects of the signal generation mechanism are still unclear or neglected. While several theoretical analyses have attempted to model signal generation with impulsive excitation [5,8,9,10], only one treatment has focused on gradual sinusoidal excitation [11]. The latter addressed the interplay of optical and thermal properties of the material in photothermal expansion, while excluding the role of geometry and mechanical properties of the probe in the signal transduction process. It is the purpose of the present analysis to fill this gap. I will do so via a simple assessment of cantilever deflection and signal generation based on the classical description of constrained expansion of a heated solid. The analysis provides guidelines for optimizing photothermal experiments with gradual excitation. The extension to the case of impulsive excitation will be left as a future development.

## 2. Analysis and Discussion

### 2.1. Analysis of Parameters Affecting Signal Generation with Gradual Excitation

The response of an AFM probe deflected by photothermal expansion under conditions of gradual excitation will be described using the classical treatment [12] of an isotropic solid undergoing constrained thermal expansion. It is assumed that the temperature increase, ΔT, and the resulting expansion, compression and deflection, are small, such that thermomechanical properties can be considered constant, and the mechanical response is elastic. The dependence of ΔT on the absorption coefficient and thermal properties of the sample, and its wavelength dependence, have been described elsewhere and will not be treated further [11]. The following analysis will be concerned with the thermoelastic properties of the sample and the probe. These are summarized in Figure 2, showing the tip-sample contact region for two possible geometries. In the simplest case a flat tip, either a cone or a truncated cylinder, is in contact with a flat sample surface (panels A, C, E). In a more complex case, closer to the common experimental situation, a curved tip is in contact with a locally flat sample surface (panels B, D, F).

Photothermal heating of the sample causes its expansion and an increase in the pressure applied at the tip-sample contact. It is assumed that only the sample absorbs light, and that heating of the tip is negligible. It is also assumed that absorbed photon energy is fully converted into a temperature increase, excluding photochemical, photoelectric and piezoelectric processes, and phase transitions. In the general case, the Young’s moduli of the sample and the tip are comparable and compression of both the sample and the tip occurs, together with a displacement of the back of the tip at the cantilever contact, δ (Panels C and D). The compression also causes a displacement of the tip-sample contact, δ′, with δ′ ≠ δ. δ′ is analogous to the indentation extracted by classical AFM measurements. Assuming an axisymmetric tip, the projection of the contact surface is a disk of radius r. In the common experimental situation where the material of the tip is much less compressible than the sample, the compression of the tip is negligible, and δ = δ′ (Panels E and F). The displacement δ tilts the cantilever of the probe, moving the laser beam across the four-quadrant detector and generating the signal (Figure 1). Maximizing the signal corresponds to maximizing the displacement, δ, which is equivalent to maximizing the deflection of the cantilever.

For a given temperature increase, the maximum deflection is determined by balancing the force from the expansion of the working medium, F_e_, with the compressive force exerted by the cantilever, F_c_. F_e_ is calculated as the force exerted by a compressible medium undergoing constrained expansion and is given by Equation (1).

F_e_ = E* α ΔT A (1)

E* is the reduced Young’s modulus. α is the thermal expansion coefficient of the sample. ΔT is the temperature difference. A is the contact area between tip and sample. Equation 1 assumes a small compression, such that shear stress at the edges of the compression area is negligible for the case of a sample larger than the tip.

Assuming a small expansion, the cantilever can be considered as a simple harmonic spring and its deflection under the action of the expansion force can be described by Hook’s Law (Equation (2)), where K is its spring constant.

F_c_ = −δ K (2)

In an experiment with gradual excitation, ΔT increases over time when the sample is irradiated at constant power, leading asymptotically to a thermal steady state when ΔT plateaus to a stable value. The maximum deflection, δ_max_, for a given temperature increase, ΔT, is obtained when F_e_ = −F_c_ (Equation (3)).

δ_max_ = (E* α ΔT A)/K (3)

If a setpoint force is being applied, the deflection of the cantilever will be different from zero in the absence of photothermal expansion. In such a case, δ is taken to be the total deflection, i.e., the sum of the setpoint deflection and the deflection caused by sample expansion, and F_c_ is equal to the sum of the setpoint force and the force from the photothermal expansion. Increasing deflection corresponds to an increasing signal, and maximizing the deflection under a set of conditions corresponds to maximizing the sensitivity of the measurement.

For the specific cases of a tip in the shape of a cylinder or a truncated cone, A is the area of the cylinder base or of the cone cross section, respectively. For a spherical tip, we approximate the contact area as the projection of the tip on the sample surface (Equation (4)), where r is the radius of the projected contact surface (Figure 2). The value of r is a function of the total force and of the properties of the probe and material. r = F_c_^1/3^{E*^−1^ RR′/(R + R′)}^1/3^ (R and R′ represent the contact area of two spheres compressed by a force F_c_. From reference [12], §9 of the second edition).

δ_max_ = (E* α ΔT πr^2^)/K (4)

The reduced modulus, E*, is defined by Equation (5).

E*^−1^ = (1 − σ^2^)/E + (1 − σ′^2^)/E′ (5)

E and E′ are the Young’s moduli of the sample and of the tip, respectively, whereas σ and σ′ are the Poisson ratios of the sample and of the tip. If the modulus of the sample is much lower than the modulus of the tip (this is the common experimental case of a hard tip and a soft sample), Equation (5) simplifies to Equation (6).

E*= E/(1 − σ^2^) (6)

Equations (3) and (4) express the dependence of δ_max_ on the thermomechanical and geometric properties of the sample and the probe. Materials with a larger thermal expansion coefficient, α, stiffer materials with larger values of E, and tips with a larger contact surface all produce larger values of deflection δ_max_. Maximum deflection decreases with increasing K, with softer cantilevers providing greater sensitivity. An important feature is the interplay of the elastic properties of both the tip and the sample, including Young’s moduli and Poisson’s coefficients, which jointly contribute to the signal. In measurements of soft matter with common AFM setups, the elastic properties of the sample are the dominant contribution to E*. However, this is not the case when the sample is a hard material, for which the joint contribution of the tip and the sample needs to be considered. Table 1 summarizes these conclusions and can be used both as a guideline for the optimization of measurements and as a reference for testing the model described in this work.

One important result of the present analysis is the rapid increase in the signal provided by larger tips-with an expanded tip-sample contact surface. Wider tips generally deliver poorer spatial resolution in scanning probe measurements, thus highlighting the existence of a trade-off between the resolution and the sensitivity of a measurement. This conclusion can better inform experimental practice, where tip selection is commonly performed with a view to its contribution to resolution, whereas other aspects are commonly neglected.

We can assess the validity of Equation (3) by comparing its predictions with this benchmark. Let us consider a sample of polystyrene (PS), for which E = 3 × 10^9^ Pa, σ = 0.35 and the linear thermal expansion coefficient α = 7 × 10^−5^ *K*^−1^ [13]. We irradiate the sample with light at the frequency of the absorption bands of PS, producing a ΔT = 1 K, a realistic value for the T increase in a polymer sample irradiated with a light source of a few 100 µW (such as from a thermal source for FTIR spectroscopy or a laser). Using a contact mode AFM probe with K = 1 N/m and the radius of the contact area r = 50 nm, Equation (3) provides a value of maximum deflection δ_max_ = 1.9 nm, close to the experimental value of 1.5 nm measured for this system [3]. Reference [3] does not report the parameters of the tip used in the experiment, and the values of K and r are based on common parameters for AFM probes for soft matter. Nonetheless, the results fall within the range allowed by the wide variability of the published values of E and α. While this is only an approximate verification, it shows that Equation (3) produces values that are plausible and consistent with available experimental results.

While the present analysis is limited to the case of gradual excitation, it is worth discussing if these conclusions can be qualitatively extended to experiments with impulsive excitation. In such a case, the amplitude of the signal is predicted to increase with the impulse of the expansion force. Therefore, quantities affecting F_e_, such as the contact area A, are also expected to similarly affect an impulsive signal. However, the extension is expected to be valid as long as no other forces (e.g., electrostatic, magnetic, photoacoustic, photoinduced) are involved in signal generation, which is not justified in the case of loss of contact between the tip and the material [14].

### 2.2. The Micromechanical Detection Scheme of Photothermal Expansion Is Described as a Photothermal Engine

To gain further insight into the processes underlying the mechanism of signal generation, I propose to complement existing descriptions with a treatment based on classical thermodynamics. The starting point of the discussion is the observation that the signal generation mechanism can be described via the thermodynamic cycle of a microscopic engine, a device that converts energy into work. Energy from absorbed photons heats the sample which, by expanding, delivers the work necessary to deflect the cantilever of the AFM probe. When the sample cools back to ambient temperature, heat is released into the environment as the cantilever returns to its resting position. Periodic excitation causes an alternating deflection and relaxation of the cantilever. The cycle is analogous to that of a classical heat engine, with two main differences, one being that the working medium (i.e., the sample) is a solid instead of a gas and the other being that the expansion stage is used to increase the potential energy of the cantilever. The differences have important consequences on the energy balance of the cycle, as discussed later.

Figure 3 shows the transformations of the sample over a full cycle of excitation and relaxation. The sample acts as the working medium of the engine, and the two terms are used interchangeably in this work. The full cycle is ideally broken down into quasi-static reversible transformations (the strokes of the engine), connecting four states described by the set of thermodynamic variables, pressure (P), volume (V) and temperature (T). State 1 is the initial resting state. In the first stroke, absorbed light delivers energy to the medium, simultaneously increasing both T and P, and bringing it to state 2. In the second stroke, the sample expands, reaching state 3. In the transition from state 2 to state 3, expansion acts against the force exerted by the cantilever and causes its deflection. In the third stroke, the expanded sample cools down to the initial temperature leading to state 4. In the fourth and last stroke, the sample is compressed back to its initial volume, corresponding to the initial state 1.

In this analysis I assume that the quantum yield for the conversion of absorbed light into heat is unity. I neglect any other radiative and non-radiative channels that may lead to dissipation of energy from absorbed photons, such as photochemical and photoelectric processes, fluorescence, and phosphorescence. Phase transitions are also excluded. I assume that the only force that acts on the AFM probe is the mechanical one arising from the action of the expanding medium. Therefore, I neglect the possibility of other interactions, such as those of electromagnetic or photoinduced origin, or short distance interactions arising from Van der Waals forces [14].

It is integral to the definition of gradual excitation that the sample expands while retaining contact with the tip of the probe. The slow operation ensures that the approximation of quasi-static reversibility can be applied to the cycle. Under conditions of gradual excitation, heating and expansion can be considered to occur simultaneously, providing a simpler cycle compared to the general one shown in Figure 3. Strokes 1–2 and 2–3 from Figure 3 can be represented as a single transformation, bringing the sample from an initial state 1 represented by T_1_, P_1_, V_1_ to a state 3 represented by T_3_, P_3_, V_3_, with T_3_ > T_1_, P_3_ > P_1_, V_3_ > V_1_. Similarly cooling and compression are simultaneous and bring the system back from state 3 to state 1. Figure 4A shows the conversion between these two states as a path in a pressure-volume (PV) diagram (solid line). The path extends along the two hyperbolas that represent isothermal transformation paths at temperatures T_1_ and T_3_ (dashed lines). During the heating and expansion stroke, from 1 to 3, heat is absorbed by the working medium in the form of energy from absorbed light (Q_in_) and work is executed by the expanding medium on the AFM probe (W_out_). During the cooling and compression stroke, from 3 to 1, heat is released by the working medium to the environment (Q_out_) and work is executed by the AFM probe on the expanding medium (W_in_) (Figure 4A). The increase in volume of the medium triggers signal transduction by deflecting the cantilever (δ). Assuming an isotropic expansion, the deflection increases monotonically with sample expansion according to δ ∝ (V_3_ − V_1_)^1/3^.

The harmonic response of the cantilever sets this mechanism apart from that of conventional heat engines. Work output from the expanding medium (W_out_) is converted into potential energy by the bending of the cantilever, instead of being transduced farther downstream. As the medium cools down and contracts, the cantilever fully returns the stored potential energy in the form of compression work (W_in_) performed on the medium. If the tip maintains contact with the sample throughout the cycle, stroke 3–1 is the exact reverse of 1–3 and total mechanical work output (W_tot_ = W_out_ − W_in_) from the engine is null when the cantilever has returned to its resting position. Because other channels for heat dissipation have been excluded, the energy provided by absorbed light has been transferred to the environment as heat and Q_in_ = Q_out_. Considering mechanical work, the process corresponds to zero engine efficiency (η), with η = W_tot_/Q_in_ and the engine is not capable of converting heat into mechanical work with cyclical operation. Nonetheless, deflection of the cantilever takes place, and provides a signal, even in the absence of net W_tot_. Figure 4B shows the deflection versus time plot for the case of a beam modulated by mechanical chopping, in agreement with the response reported experimentally [3].

Neither W_out_ nor η relate directly to the intensity of the signal, which is determined by the deflection of the cantilever. Therefore, the quantities that are normally used to characterize the performance of classical engines appear to have no direct consequence on the performance of a measurement under gradual excitation. Although this conclusion may appear to be a discrepancy, the discrepancy is only apparent. In contrast to a classical engine, which aims to convert heat into mechanical work, the purpose of this photothermal engine is analytical. The movement of the engine in response to the presence of an absorbing sample is converted to a voltage difference at the four-quadrant detector, which in turn provides information on the presence or absence of an absorbing material. The energy involved in signal generation from the four-quadrant detector is the one provided by the AFM probe laser, not from the photothermal micro engine. In this context, the latter mechanism acts as a machine that processes information, while not providing any usable work output.

## 3. Conclusions

I have described a detection scheme of photothermal expansion in terms of a light-driven micromechanical engine. I have used a classical treatment of constrained thermal expansion to provide a theoretical description of signal generation in photothermal measurements with gradual excitation. The analysis is based on classical thermodynamics and is valid as long as materials can be described by their bulk properties. The analysis highlights the dependence of the signal on the thermomechanical and geometrical properties of tip and sample. I note a linear dependence of the signal on the contact area of tip and sample, which implies a trade-off between the spatial resolution provided by the AFM tip and signal intensity. The results support the viability of this configuration for quantitative studies of the thermal and mechanical properties of microscopic samples and for their spectroscopic characterization. While the model has been developed assuming isotropic solid materials, it can be extended to liquid and gaseous materials for the specific case in which the material is enclosed in a perfectly rigid container with a moving, but tightly sealed end. This includes the case of the classical engine, with a working fluid enclosed inside a cylinder capped by a piston.

While interest in this mechanism was driven by its potential spectroscopic applications, as a variation of the AFM-IR experiment, it must be stressed that its utility is more general, as it allows quantitative measurement of multiple properties (as defined by Equation (3)) on microscopic samples, including temperature variations. The lower limit for the spatial resolution of the measurement depends on the size of the probe-sample contact and on the thermoelastic properties of sample and probe. Spatial resolution below one micrometer can be easily achieved with components used in routine AFM measurements.

In the case of gradual excitation, the micromechanical system can be described by a thermodynamic cycle operating under approximately quasi-static conditions, and can be considered to be close to the reversibility limit. Unlike classical heat engines, this cycle does not produce any useful mechanical work on the environment and has zero efficiency. However, the lack of efficiency does not hinder its analytical function, which involves the production and processing of information concerning the sample material.

## Figures and Tables

**Figure 1:**
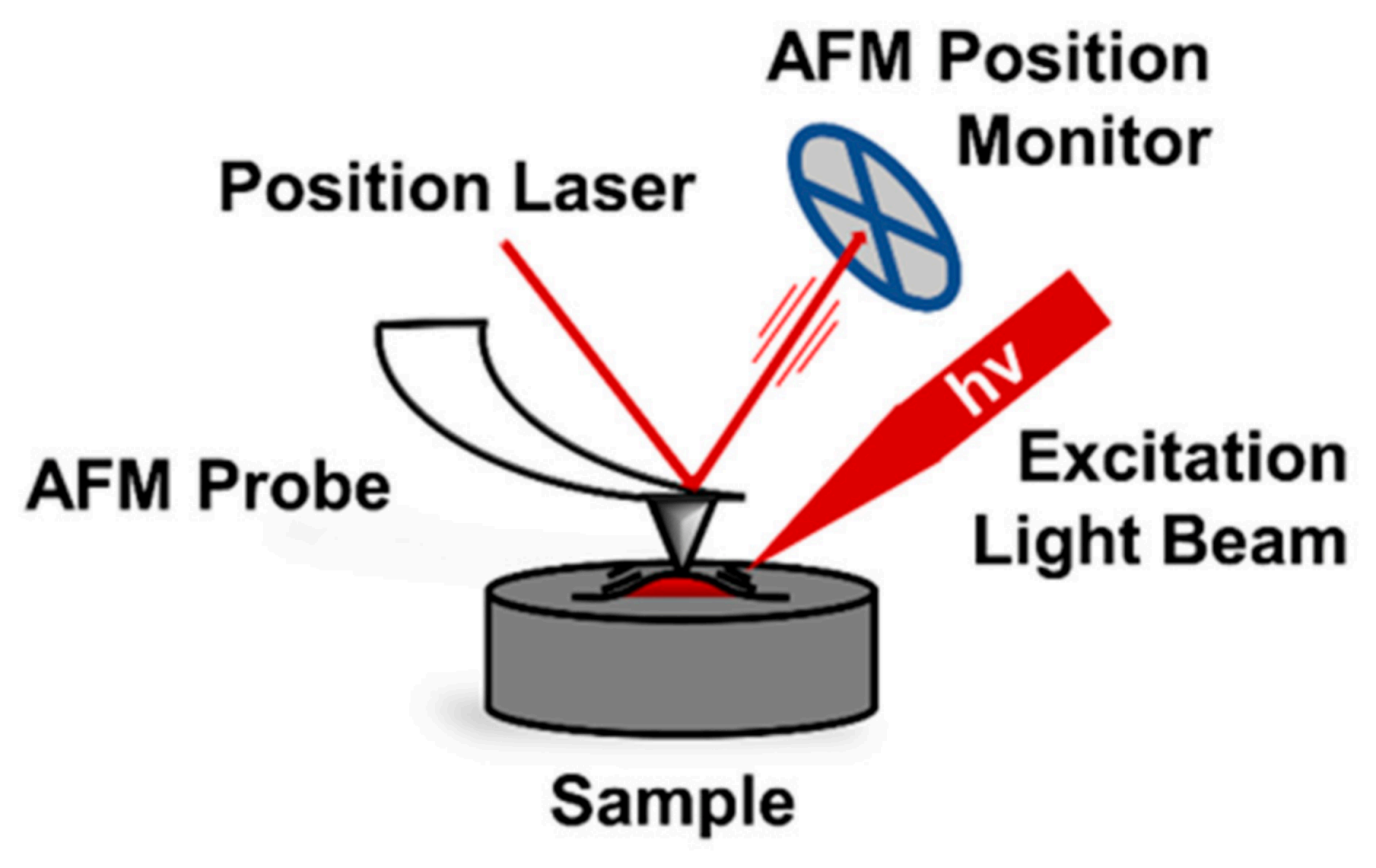
Micromechanical detection of photothermal expansion. The sample is mounted as for a conventional AFM experiment, and irradiation occurs by focusing light at the tip-sample contact region. An absorbing material expands upon illumination, and the expansion is recorded via the deflection of the cantilever and quantified by the movement of the AFM monitor laser on a four-quadrant detector.

**Figure 2:**
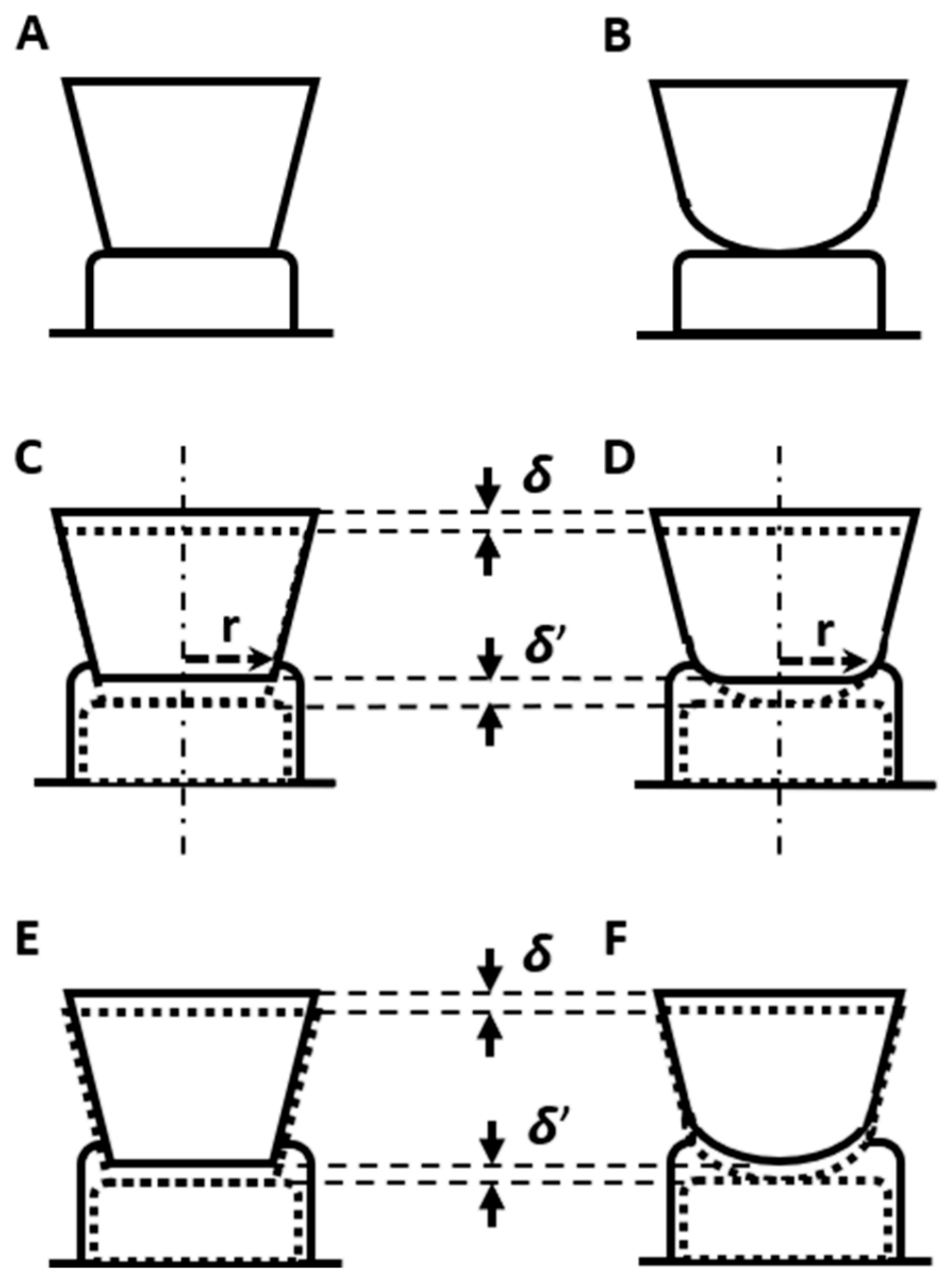
The tip-sample contact region in the measurement of photothermal expansion for the case of a flat tip (Panels (**A**,**C**,**E**)) or a curved tip (Panels (**B**,**D**,**F**)). (**A**,**B**) Resting position of the tip in contact with the sample before excitation. (**C**,**D**) Photothermal expansion of the sample when tip and sample have similar rigidity (i.e., the Young’s moduli are comparable). The dotted profiles show the position of sample and tip before thermal expansion takes place. The continuous profiles show the equilibrium position after expansion. The expansion causes compression of both the tip and the sample at the contact region, defining a contact surface of radius r. The contact is displaced by δ′ from the initial position, while the back of the tip, at the cantilever contact, is displaced by δ. In the general case, δ ≠ δ′. (**E**,**F**) Photothermal expansion of the sample when the sample is much more compressible than the tip (i.e., the Young’s modulus of the sample is much smaller than that of the tip). The dotted profiles show the position of sample and tip before thermal expansion takes place. The continuous profiles show the equilibrium positions after expansion. The expansion causes compression of the sample at the contact region, defining a contact surface of radius r. The tip is displaced without being deformed. In this case, the displacement of the contact surface is the same as the displacement of the back of the tip, δ = δ′.

**Figure 3:**
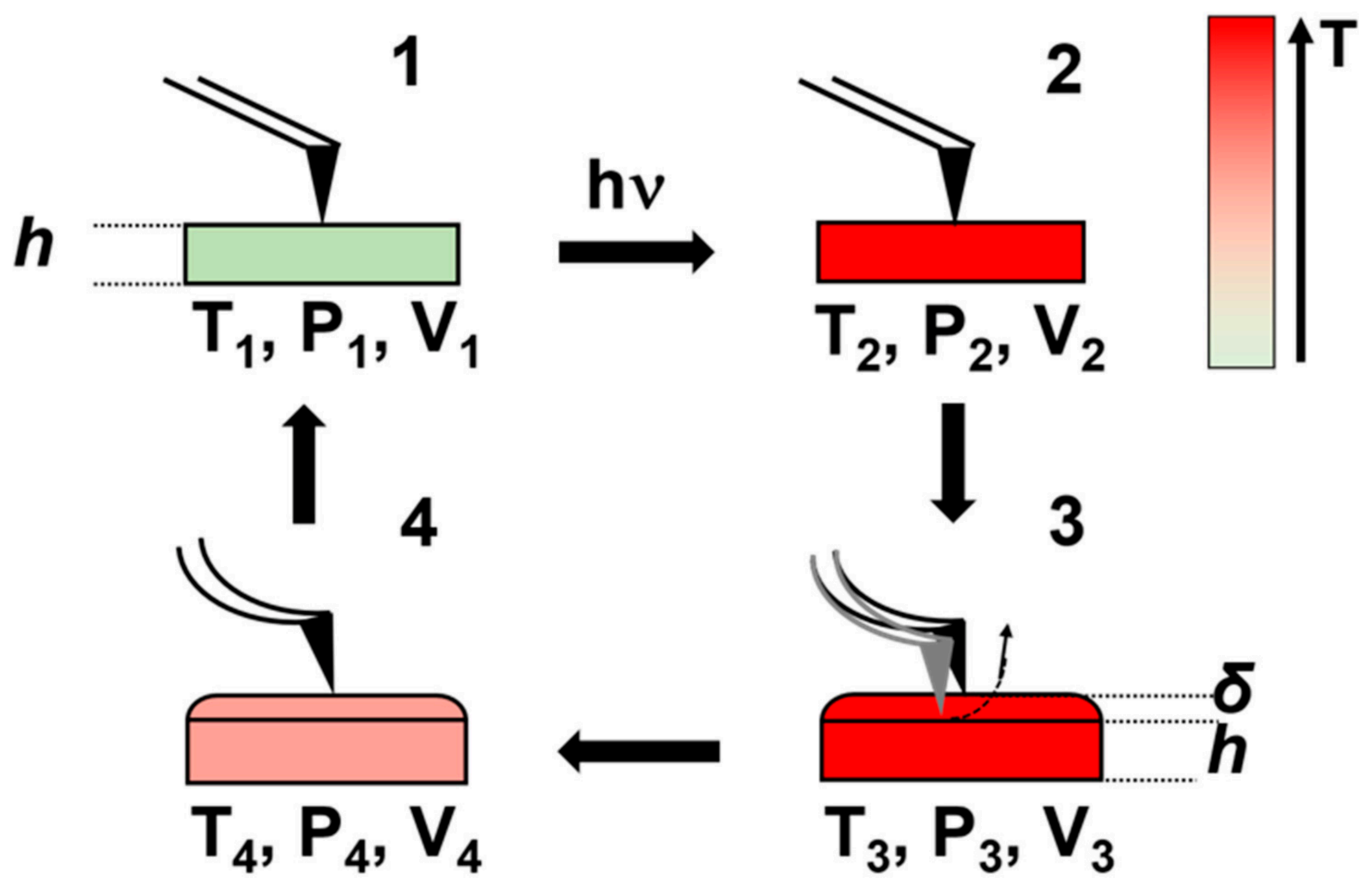
Elementary states and transformations (steps) of the micromechanical engine cycle. T_n_, P_n_, V_n_: respectively, temperature, pressure, and volume of state n. d: sample thickness. δ: expansion/cantilever deflection. h: sample thickness at rest. Red color graphically indicates higher temperature. 1—Initial state (resting state). 2—After the temperature increase caused by light absorption. 3—After expansion from state 2. 4—After cooling of state 3.

**Figure 4:**
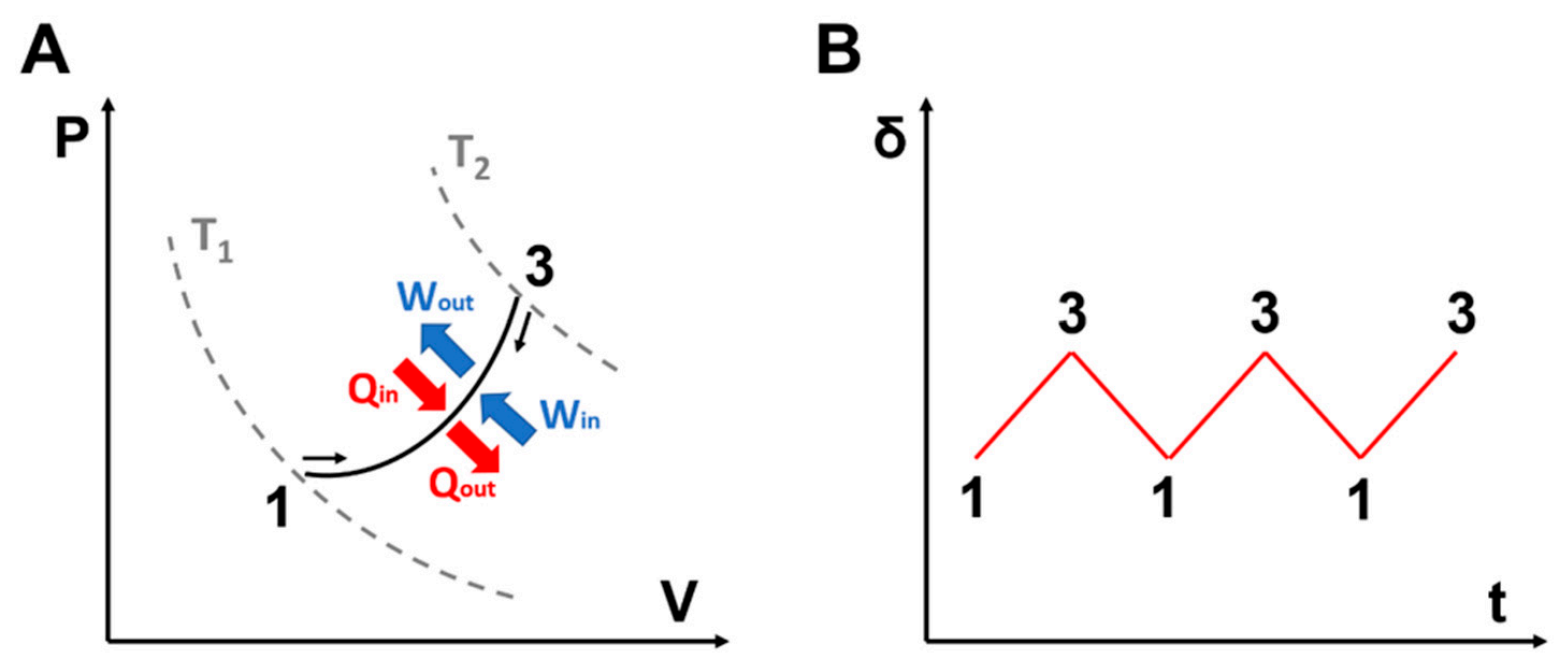
The photothermal engine cycle under conditions of gradual excitation. (**A**) Engine cycle in a PV diagram. The medium absorbs light, progressing from state 1 to state 3, with a corresponding increase in pressure, volume, and temperature. Interruption of illumination leads to cooling and decompression of the sample, returning to state 1 by reversing the expansion stroke. (**B**) Deflection (δ) vs. time (t) diagram corresponding to the cyclical repetition of the two strokes in panel (**A**).

**Table 1:** Effect of sample and probe properties on the deflection signal from photothermal expansion. The “+” sign indicates quantities that correlates positively with an increased signal, while the “−” sign indicates quantities that correlate negatively. K: cantilever force constant. A: tip/sample contact surface. E, E′: Young’s Moduli of sample and tip. α: thermal expansion coefficient of sample. σ, σ′: Poisson ratios of tip and sample. r: radius of tip/sample contact surface. ΔT: photothermal-induced temperature increase.

Quantity	Predicted Correlation with Deflection
K	−
A, r	+
E, E′	+
α	+
σ, σ′	+
ΔT	+

## Data Availability

No new data were generated for this work.

## Abbreviations

The following abbreviations are used in this manuscript:

AFM-IR	Atomic Force Microscopy Infrared
AFM	Atomic Force Microscopy
CW	Continuous Wave
FTIR	Fourier Transform Infrared
PTIR	Photothermal-Induced Resonance
PS	Polystyrene
